# CSNK1E is involved in TGF-β1 induced epithelial mesenchymal transformationas and related to melanoma immune heterogeneity

**DOI:** 10.3389/fphar.2024.1501849

**Published:** 2025-01-13

**Authors:** Wangbing Hong, Xin Wang, Xinyu Huang, Pengfei Chen, Yifan Liu, Ziying Zheng, Xin You, Yinghua Chen, Zengxin Xie, Gongnan Zhan, Heping Huang

**Affiliations:** ^1^ Department of Plastic and Cosmetic Surgery, Jiangxi Maternal and Child Health Hospital, Nanchang, Jiangxi, China; ^2^ Medical Center of Burn Plastic and Wound Repair, The First Affiliated Hospital of Nanchang University, Nanchang, Jiangxi, China; ^3^ Department of Plastic and Aesthetic Surgery, Nanfang Hospital of Southern Medical University, Guangzhou, Guangdong, China

**Keywords:** CSNK1E, TGF-β 1, epithelial mesenchymal transformationa, melanoma, LASSO

## Abstract

**Introduction:**

Melanoma (MM), the deadliest form of skin cancer, originates from melanocytes. Despite advances in immunotherapy that have somewhat improved the prognosis for MM patients, high levels of resistance to treatment continue to result in poor clinical outcomes. Identifying novel biomarkers and therapeutic targets is critical for improving the prognosis and treatment of MM.

**Methods:**

In this study, we analyzed the expression patterns of WNT signaling pathway genes in MM and explored their potential mechanisms. Using Cox regression analysis, we identified 19 prognostic-related genes. Consistency clustering was performed to evaluate the potential of these genes as classifiers for prognosis. The Least Absolute Shrinkage and Selection Operator (LASSO) algorithm was then applied to refine the gene set and construct a 13-gene prognostic model. We validated the model at multiple time points to assess its predictive performance. Additionally, correlation analyses were performed to investigate the relationships between key genes and processes, including epithelial-to-mesenchymal transition (EMT) and immune responses.

**Results:**

We identified that CSNK1E and RAC3 were significantly positively correlated with the EMT process, with CSNK1E showing a similar expression trend to EMT-related genes. Both genes were also negatively correlated with multiple immune cell types and immune checkpoint genes. The 13-gene prognostic model demonstrated excellent predictive performance in MM prognosis. Pan-cancer analysis further revealed heterogeneous expression patterns and prognostic potential of CSNK1E across various cancers. Wet experiments confirmed that CSNK1E promotes MM cell proliferation, invasion, and migration, and enhances malignant progression through the TGF-β signaling pathway.

**Discussion:**

Our findings suggest that CSNK1E plays a crucial role in MM progression and could serve as a potential therapeutic target. The WNT and TGF-β pathways may work synergistically in regulating the EMT process in MM, highlighting their potential as novel therapeutic targets. These insights may contribute to the development of more effective treatments for MM, particularly for overcoming resistance to current therapies.

## 1 Introduction

Melanoma (MM) is the deadliest form of skin cancer (Guo et al., 2021), accounting for over 75% of skin cancer-related deaths (Rebecca et al., 2020) and approximately 0.7% of all cancer mortality (Schadendorf et al., 2018). Moreover, MM is among the few cancers whose incidence is currently on the rise (Poklepovic and Luke, 2020).

Melanoma incidence and mortality are higher in men than in women, and the underlying biological mechanisms responsible for the sex differences in cutaneous melanoma are unknown and complicated by clinical variables such as anatomical site, skin light type, body mass index, and variability in immune response. Therefore, we sought to investigate prognostic and immunological differences in melanoma by sex.

Melanocytes originate from neural crest stem cells (NCSC), and their malignant transformation leads to MM. Typically, MM arises from nevus and/or intermediate lesions, undergoing progressive dysplasia before becoming invasive and ultimately metastatic (Lin and Fisher, 2007). The transformation of melanocytes into MM is primarily driven by carcinogenic signaling pathways, which are triggered by a combination of environmental and genetic factors. Common environmental factors include ultraviolet (UV) exposure in Caucasians, whereas in individuals of Asian and African descent, trauma, chronic inflammation, and infections are more prevalent triggers (Liu et al., 2016; Splendiani et al., 2024). Genetic factors often involve a relevant family history (Splendiani et al., 2024). Phenotypic heterogeneity exists within MM, which can significantly affect diagnosis and prognosis (Grafanaki et al., 2023). Studies have classified MM into four subtypes based on driving mutations: BRAF-mutant, RAS-mutant, NF1-mutant, and wild-type BRAF/RAS/NF1, with common mutations also including KIT or GNAQ/GNA11 (Kiuru and Busam, 2017). Additionally, transcriptomic analyses have categorized MM into: undifferentiated (AXL-high, SOX10/NGFR/MITF-low), neural crest-like (SOX10-high, NGFR-high, MITF-low), transitory (SOX10-high, NGFR-medium, MITF-medium), and melanocytic (SOX10-high, NGFR-low, MITF-high) (Comandante-Lou et al., 2022). Histopathologically, MM is generally classified into superficial spreading, nodular, malignant lentigo, and acral lentiginous types, which may correspond to distinct pathogenic mechanisms, thus influencing treatment approaches. For instance, UV exposure may drive BRAF mutations, often resulting in superficial spreading MM (Armstrong and Cust, 2017), while trauma and inflammation can elevate cytokines and reactive oxygen species, significantly correlating with acral MM (Zhang et al., 2014). A comprehensive grasp of the molecular mechanisms driving MM has advanced the creation of targeted therapies. Research indicates that immune checkpoint inhibitors are effective in approximately one-third of patients (Sharma et al., 2017). BRAF inhibitors (BRAFi), as well as combinations of BRAFi and MEK inhibitors (MEKi), can benefit up to 50% of BRAF-mutant patients with advanced MM (Flaherty et al., 2012). Furthermore, combined checkpoint inhibitors, such as anti-PD-1 and anti-CTLA-4 antibodies, can improve overall survival in advanced patients (Rogiers et al., 2019). Talimogene laherparepvec, as the first approved oncolytic virus therapy, has also shown survival benefits. However, over 80% of patients experience recurrence after BRAF/MEK inhibitor treatment, and the efficacy of targeted therapies in wild-type BRAF patients is limited (Johnpulle et al., 2016), with 60%–70% of patients not responding to checkpoint inhibitor therapy (Jerby-Arnon et al., 2018). Therefore, it is essential to further explore the molecular mechanisms involved in MM development and to identify key target genes to enhance treatment and prognostic evaluation.

The WNT signaling pathway comprises 19 glycoproteins, including β-catenin, Disheveled (DVL), Lrp6, and Axin (Bryja et al., 2017). This pathway is involved in regulating the cell cycle and embryonic development, and it plays significant roles in inflammation and cancer progression (Clevers, 2006). Recent studies suggest that the WNT pathway could serve as a biomarker and a potential therapeutic target in cancer (Miete et al., 2022). There are two main classes into which the WNT pathway is categorized: the canonical pathway and the non-canonical pathway (Akoumianakis et al., 2022; Liu et al., 2022; Zhao et al., 2022). The canonical WNT/β-catenin pathway is linked to the nuclear translocation of β-catenin and usually plays a role in the proliferation and maintenance of stem and progenitor cells (Tai et al., 2015). In contrast, the non-canonical WNT pathways may relate to β-catenin-independent mechanisms (Zimmerman et al., 2012) and participate in regulating planar cell polarity (PCP) signaling and WNT/Ca^2+^ signaling pathways (Anastas and Moon, 2013). The PCP signaling pathway modulates cytoskeletal remodeling, cell polarity regulation, and cell migration (Logan and Nusse, 2004; Semenov et al., 2007), whereas the WNT/Ca^2+^ signaling pathway influences cancer progression and intercellular communication (Liang et al., 2003; Vargas et al., 2019). Alterations in the WNT signaling pathway are observed in many cancers. Studies suggest that in breast cancer, the composition of WNT signaling proteins is modified, with alterations observed at the DNA level, in mRNA post-transcriptional modifications, and in protein post-translational modifications. Nevertheless, the activation of WNT signaling is mainly driven by epigenetic changes (Xu et al., 2020). In colorectal cancer (CRC), the WNT/β-catenin pathway is crucial for both the initiation and sustenance of the disease, with suppression of WNT pathway expression demonstrating therapeutic potential against CRC (Zhao et al., 2022). Additionally, the abnormal activation of WNT/β-catenin signaling may be associated with the development of prostate, breast, ovarian, and pancreatic cancers (Jung and Park, 2020). Many surface markers of cancer stem cells serve as targets for the WNT pathway, and when this pathway is dysregulated, it can result in resistance to tumor treatment (Ring et al., 2014). These factors suggest that the WNT pathway significantly influences the occurrence, development, and prognosis of various tumors. Currently, multiple studies have elucidated the role of the WNT/β-catenin pathway in malignant melanoma (MM), but consensus has not been reached. Activated canonical WNT/β-catenin signaling has been associated with reduced melanoma proliferation, acting as a negative regulator of tumor growth in both patient-derived tissues and mouse models of melanoma (Kim et al., 2020). However, other studies have shown that WNT signaling is reactivated during the malignant transformation of melanoma (Sinnberg et al., 2018). Aberrant activation of the WNT/β-catenin pathway has been observed in nearly one-third of human melanoma cases (Vaid et al., 2016). Despite the conflicting findings regarding the WNT pathway in MM, its diverse roles in cancer underscore the necessity for further investigation into its specific functions in MM. Such research could provide valuable insights for developing novel therapeutic strategies.

In this study, we aimed to explore potential therapeutic targets of the WNT pathway in MM through bioinformatics analysis. Initially, we performed a Cox regression analysis on the WNT pathway gene set, identifying 19 genes. Subsequently, we conducted consistent clustering analysis on these 19 genes, resulting in the identification of two subtypes. We then constructed a model using these genes and selected 13 that were prognostically relevant. Using the model, we predicted risk scores for high-risk and low-risk groups and analyzed the expression levels of the 13 genes within both groups, leading to the identification of two epithelial-mesenchymal transition (EMT) -related genes, CSNK1E and RAC3. We conducted a further analysis of the relationship between these genes and immune cells, particularly noting the relationship between CSNK1E and immune checkpoints. Finally, we performed a pan-cancer analysis of the CSNK1E gene, investigating its expression across various cancers and its prognostic implications, as well as its co-expression with EMT-related genes. Our work offers new targets for MM research and provides robust support for both scientific and clinical studies. What’s more, We conducted three phenotypic experiments following the knockdown of CSNK1E in human melanoma cell lines to enhance the credibility of our bioinformatics conclusions.

## 2 Materials and methods

### 2.1 Data acquisition and preprocessing

We downloaded the dataset GSE91061 from the Gene Expression Omnibus (GEO, https://www.ncbi.nlm.nih.gov/geo/) website. After integrating the data, we converted it into Transcripts Per Million (TPM) format and applied log_2_ transformation to mitigate excessive data dispersion. GEO is an open-access database that does not require additional ethical approval. We adhered to relevant guidelines for data collection and utilization.

### 2.2 Gene screening and consistency clustering

We conducted a Cox regression analysis on 5,917 genes related to the WNT pathway in MM. Genes were deemed significant if they met the criteria of *p* < 0.05 and a hazard ratio (HR) not equal to 1, indicating their impact on survival in MM patients. A forest plot was generated using the “forestplot” package to visualize these results, allowing us to identify genes influencing prognosis based on their HR. For comparative analysis, we performed consistency clustering on the selected genes using the R package “ConsensusClusterPlus”. The optimal number of clusters (k) was established by identifying the value where the cumulative distribution function (CDF) curve levels off, signifying maximum stability without any significant increases. We further validated this k value using a Delta Area Plot, typically choosing the last inflection point as the optimal cluster number. Visualization of the results was accomplished with “ggplot2,” revealing a consistency heatmap that illustrated the “high cohesion, low coupling” characteristics of the clusters. Finally, we utilized the R package “survival” to conduct survival analyses on the identified clusters, employing the “ggsurvplot” function from the “survminer” package to visualize survival outcomes between different clusters.

### 2.3 Model construction and risk assessment

To further identify Wnt pathway genes associated with prognosis, we employed the Least Absolute Shrinkage and Selection Operator (LASSO) method to screen and construct a relevant prognostic model. The optimal model fit is established by identifying the minimum likelihood deviation on the *y*-axis of the cross-validation curve, which signifies the best log(λ) value. Following this, we included the variables related to this optimal log(λ) value in the equation. The risk score for each patient was calculated by summing the products of the coefficients and expression levels of the respective variables (genes). The GSE91061 cohort data was divided into high-risk and low-risk groups based on the median score for risk assessment. Utilizing the R package “ggrisk”, we demonstrate how patients’ survival times and the expression levels of the model genes change as the risk score increases. Following that, survival analysis was performed on both risk groups, utilizing the “ggsurvplot” package to visualize the survival curves. Additionally, we assessed the prognostic prediction efficacy of the model for two risk groups using receiver operating characteristic curve (ROC), where an area under the curve (AUC) value greater than 0.6 indicates better performance. Finally, we visualized the correlation between the model genes and EMT-related physiological processes using the “ggplot2” package.

### 2.4 Survival analysis and immune-related analysis

Firstly, we conducted survival analyses on the model genes. The “ggsurvplot” function from the R package “survminer” was employed to visualize the survival curves. To explore the connection between gene expression levels and immune cell infiltration, we utilized “ggplot2,” creating lollipop plots that demonstrated the correlation between the two selected genes and immune cells. A significant and strong correlation between the two variables is considered when *p* < 0.05 and the |R| > 0.2. A positive R value indicated a positive regulatory relationship between the gene and immune cells, while a negative R value suggested a negative regulatory relationship.

Subsequently, we selected the four types of immune cells most strongly correlated with these two genes and visualized the relationships using scatter plots. The statistical significance was established at *p* < 0.05, with the magnitude of R reflecting the strength of correlation. Based on the outcomes of these correlation analyses, we further explored immune checkpoint genes related to CNSK1E using the “IOBR” package for correlation analysis of gene expression data. This yielded several immune checkpoint genes significantly correlated with CNSK1E expression, which were visualized as boxplots using the “ggpubr” package.

### 2.5 Pan-cancer analysis

We analyzed the expression levels of CSNK1E across 33 cancer types and compared them with normal control groups, utilizing the “ggpubr” package for visualization to elucidate the potential role of CSNK1E in cancer development. We then investigated the effect of the CSNK1E gene on overall survival (OS) in these cancers, treating results as statistically significant when *p* < 0.05. Prognostic relevance was assessed based on the log_10_ (HR) values: a log_10_ (HR) > 0 indicated that abnormal CSNK1E expression may correlate with poorer survival rates, while log_10_(HR) < 0 suggested a potential association with better survival rates. Further, we analyzed the co-expression of CSNK1E with nine genes related to EMT to investigate the relationship between CSNK1E and EMT, hypothesizing potential functions of CSNK1E that could accelerate the discovery and functional analysis of new genes. Finally, we conducted a dry analysis on 37 different cancer types, identifying statistical significance at *p* < 0.05. To assess the strength of the association with stem cells, the Pearson correlation coefficient was used, where higher coefficients reflect stronger correlations. This was visualized using “ggplot2” to examine the similarities between tumor cells and stem cells.

### 2.6 Tissue acquisition from patients

We selected melanoma and normal tissue samples from six patients diagnosed with melanoma at Southern Hospital. All selected patients received a definitive diagnosis of melanoma, with other diseases excluded, and none had undergone any treatment prior to sampling. Informed consent was secured from all patients to safeguard their privacy and rights. The ethics committee of Southern Hospital approved our experimental ethics documents.

### 2.7 Cell culture and transfection

For the wet lab validation of our results, we utilized human melanoma cell lines COLO 792, COLO 829, SK-MEL-3, Hs 939. T, and A-375, along with the normal human skin cell line TE353. sk, all sourced from the Chinese Academy of Sciences Cell Bank. COLO 792 and COLO 829 were cultured in Roswell Park Memorial Institute 1,640 (RPMI-1640, HyClone, United States), while SK-MEL-3, Hs 939. T, A-375, and TE353.sk were cultured in Dulbecco’s Modified Eagle Medium (DMEM, HyClone, United States). All media contained 10% fetal bovine serum (FBS, KeyGEN, China) and 1% penicillin-streptomycin mix (Procell, China), with cell culture flasks incubated at 37°C in 5% CO_2_. The medium was replaced every 36 h to maintain the cells in a good logarithmic growth phase.

We conducted transfection experiments for the cell lines COLO 792 and COLO 829. To inhibit the expression of the gene CSNK1E in the cell lines, we commissioned a biotech company to design and produce siRNA and shRNA (Sangon Biotech, China) for knocking down CSNK1E, using a negative control (NC) as a comparison. Trypsin (KeyGEN, China) was used to digest the cells, which were then thoroughly resuspended in the culture medium. Following this, the cells were evenly distributed into a 6-well plate at a density of 3 × 10^4^ cells per well, with each well adjusted to a total volume of 2 mL using the medium. After observing cell adhesion under the microscope, siRNA was mixed with the transfection reagent Lipofectamine^TM^ 3,000 (Thermo, United States) in a specified ratio and allowed to sit at room temperature for 10 min as per the instructions. The mixture was then added to the wells using a micropipette. During transfection, the medium was replaced every 5 h, and experiments were carried out 48 h after the transfection was completed. The sequences of the shRNAs used in our study are as follows (5′-3′):

sh-Negative control: UUC​UCC​GAA​CGU​GUC​ACG​U

sh- CSNK1E-1: CUUAGUGUCUUCAUGUAU

sh- CSNK1E-2: AGC​GGG​UCC​UUC​GGA​GAU.

### 2.8 Western blot assay

First, we extracted protein from both tissue and cell samples. For patient and normal control tissues, we added protein lysis buffer (Beyotime, China, RIPA lysis buffer: protease inhibitor = 100:1) to the pre-weighed tissues, minced them on ice, and subjected them to ultrasonic disruption. For the cell lines, the cells in the 6-well plate were digested and transferred to centrifuge tubes, centrifuged at 800 rpm for 5 min, and the supernatant was discarded. The protein lysis buffer was added to the cell pellet, and the mixture was thoroughly mixed using a pipette. Both the disrupted tissue and cell mixtures were lysed on ice for 30 min, with gentle mixing every 10 min. Subsequently, they were centrifuged at 12,000 rpm at 4°C for 15 min, and the supernatant was retained. Next, we determined the protein concentration using the BCA method, performed in a 96-well plate with three replicates for each sample group. Each well received 2 µL of the protein sample, 18 µL of PBS, varying concentrations of protein standard solutions, and 200 µL of BCA working solution (Beyotime, China), followed by incubation at 37°C for 30 min. Absorbance at 562 nm was then measured using a microplate reader to determine the concentrations of the protein samples and to estimate the loading amounts for electrophoresis. Each lane was prepared by mixing the sample with loading buffer (Beyotime, China) and PBS in a specified ratio, heated in a 95°C water bath for 5 min to denature the proteins, and then cooled on ice. The sample proteins were subjected to SDS-PAGE electrophoresis at 150V, which was stopped after approximately 1 h. The membrane transfer was subsequently carried out by assembling the apparatus and adding the transfer buffer, followed by setting a current of 200 mA to transfer the proteins onto a PVDF membrane. The PVDF membrane was placed in an incubation box, where blocking solution was added and the membrane was incubated on a shaker at room temperature for 15 min. Dilute the primary antibody (Polyclonal antibody, Proteintech, United States) and add it to the incubation box, then shake overnight at 4°C. Subsequently, introduce the diluted secondary antibody (HRP-conjugated Goat Anti-Rabbit IgG (H + L), Cat No: SA00001-2, Proteintech, United States) and incubate at room temperature for 1.5 h. Prior to the addition of the blocking solution, primary antibody, and secondary antibody, as well as after the incubation, wash the PVDF membrane three times with TBST (KeyGEN, China), allowing 5 min between each wash. Finally, apply chemiluminescent substrate to the PVDF membrane, expose it using a luminescence imaging system, and quantify the protein band intensity using ImageJ software.

### 2.9 Colony formation assay

After 48 h of transfection, we performed a colony formation assay on the cell lines COLO 792 and COLO 829. The cells from the original six-well plate were digested with trypsin and seeded into a new six-well plate, with 700 cells per well. The new six-well plate was placed in a 37°C incubator with 5% CO_2_ to continue cell culture, with media changes and cell observations every 72 h. Cultivation was stopped and images were taken when it was observed under a microscope that the majority of individual clones contained more than 50 cells. After washing with PBS, 1 mL of paraformaldehyde (Solarbio, China) was added to each well to fix the cells for 30 min, followed by the addition of 1 mL of crystal violet staining solution (Solarbio, China) to each well for cell staining. After 40 min, the cells were rinsed multiple times with PBS and then left to dry. Finally, photographs were taken of the entire six-well plate and each individual well, and the cells were counted.

### 2.10 Wound healing assay

We conducted a scratch assay on the COLO 792 and COLO 829 cell lines 48 h post-transfection. Cells were placed in a 24-well plate, with the culture medium being changed every 6 h. A 200 µL pipette tip was used to gently and uniformly create a linear scratch in each well, assisted by a ruler, followed by PBS washing to remove floating cells. Images of the wells were captured at this point (designated as time zero) to record the wound area. A basic medium without FBS was then added, and the plate was incubated at 37°C. After an additional 48 h, we captured images again to assess wound healing and document the wound area at the 48-h mark. Furthermore, for the COLO 792 cell line, we also investigated the effects of varying concentrations of TGF-β1 (0 ng/mL, 10 ng/mL, and 20 ng/mL) on cell migration capabilities, as well as the impact of knocking down CSNK1E on high-concentration TGF-β1 (20 ng/mL) induced cell migration.

### 2.11 Transwell assay

To assess the invasion and migration capabilities of the cells before and after CSNK1E knockdown, we conducted a transwell assay on the COLO 792 and COLO 829 cell lines, 48 h following transfection. The cells were digested with trypsin and resuspended in serum-free medium. Chambers (Corning, United States) were placed in each well of a 24-well plate, and the cells were evenly seeded into the chambers at a density of 4 × 10^4^ cells per well. Each chamber was filled with a total volume of 200 µL of serum-free medium, while 600 µL of FBS-rich medium was added outside the chambers. The 24-well plate was then incubated at 37°C for 24 h. Following this, the media inside and outside the chambers were discarded, and the chambers were washed with PBS. After adding paraformaldehyde to the wells, the chambers were fixed at room temperature for 20 min. Crystal violet staining was performed in the dark for 20 min, followed by PBS washing, and any remaining cells inside the chambers were scraped off using moist cotton swabs. High-power fields (200× magnification) of the chambers and wells were captured under a microscope, and cell counts were facilitated using ImageJ software. We evaluated the cells’ migration and invasion capabilities in succession through the transwell assay. n preparation for the invasion assay, the chambers were pre-coated with Matrigel (Corning, United States) before seeding the cells; however, this step was omitted for the migration assay. Additionally, for the COLO 792 cell line, we investigated the effects of different concentrations (0 ng/mL, 10 ng/mL, and 20 ng/mL) of TGF-β1 on cell invasion and migration, as well as the impact of high-concentration TGF-β1 (20 ng/mL) following CSNK1E knockdown.

### 2.12 Immunofluorescence assay

To examine the alterations in protein levels of CSNK1E, Vimentin, and ZO1 after CSNK1E knockdown, we conducted an immunofluorescence assay on the COLO 792 cell line. After 48 h of transfection, the cells were digested with trypsin and seeded into a 12-well plate, replenishing the well volume with culture medium to allow for adhesion. Following a wash with PBS, the cells were fixed with formaldehyde for 30 min and then washed three times with PBS. Subsequently, 0.2% Triton X-100 (Gibco, United States) was added to permeabilize the cell membrane at room temperature for 5 min. Afterward, a blocking solution (Gibco, United States) was added, which was removed after 60 min using a pipette. The diluted primary antibody (Proteintech, United States) was then introduced to the wells, and the 12-well plate was incubated overnight at 4°C on a shaker. After washing with PBS to remove unbound primary antibody, the diluted secondary antibody (CoraLite488-conjugated Goat Anti-Rabbit IgG (H + L), Cat No: SA00013-2, Proteintech, United States) was added and incubated at room temperature for 40 min, with unbound secondary antibody also washed away with PBS. Finally, DAPI fluorescent dye (SINOPHARM, China) was applied to the wells and incubated in the dark for 5 min to label the cell nuclei. The results were examined, and images were taken with a fluorescence microscope.

### 2.13 Statistical analysis

All statistical analyses were conducted using R software (version 4.1.3). Unless otherwise specified, our figures were generated using the “ggplot2” package. A *p*-value less than 0.05 was considered statistically significant (*p < 0.05; **p < 0.01; ***p < 0.001; ****p < 0.0001).

## 3 Result

### 3.1 Gene screening and consistency clustering

**FIGURE 1:**
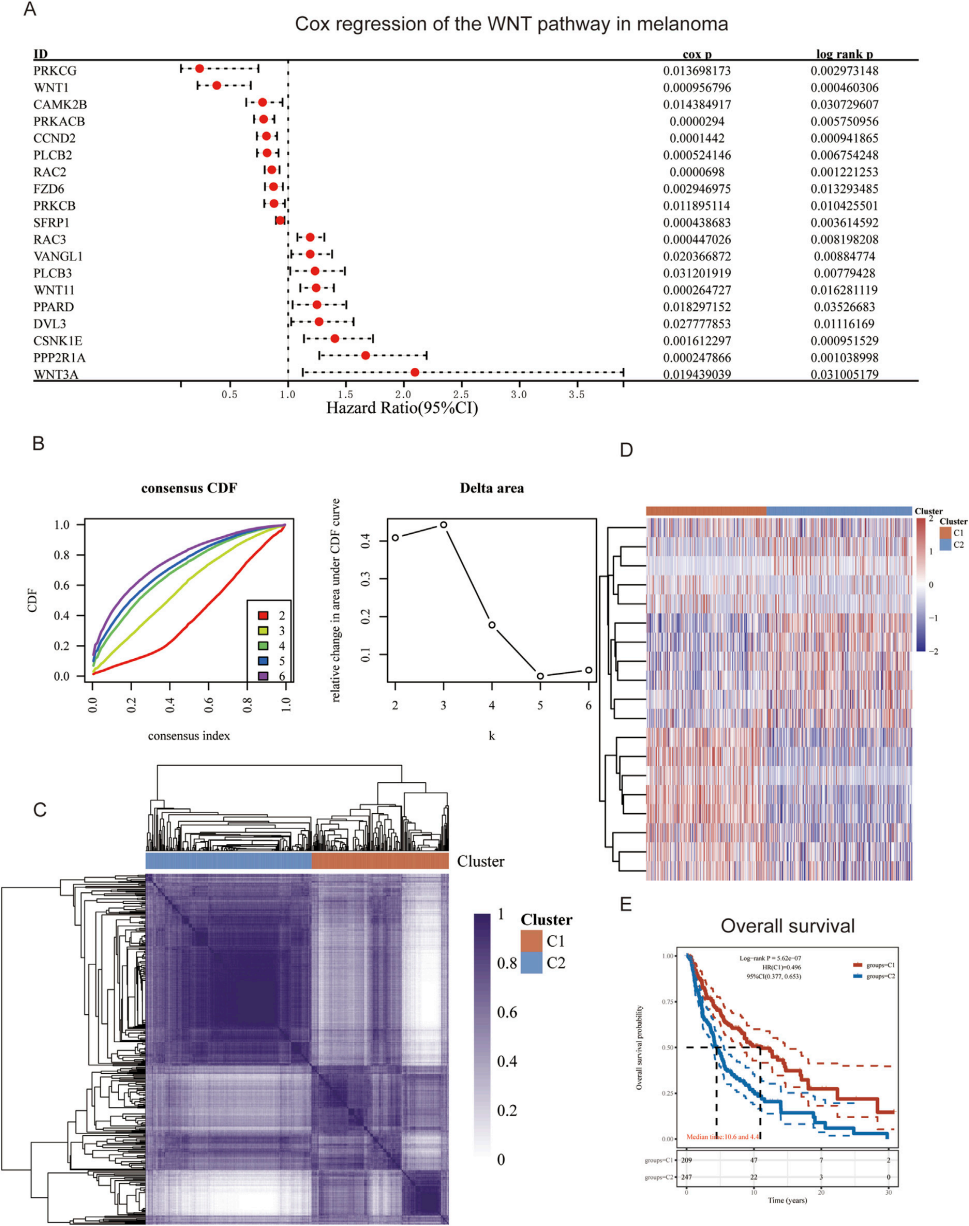
Gene Screening and Consistency Clustering. **(A)** Forest plot of Cox regression analysis for WNT pathway gene set; **(B)** Consistency cumulative distribution function and Delta area plot; **(C)** Consistency heatmap with two clusters; **(D)** Heatmap of differentially expressed genes; **(E)** Survival curves for patient groups C1 and C2.

We initially conducted a Cox analysis on 5,917 genes involved in the WNT pathway, identifying 19 genes including PRKCG and WNT1. Among these, 10 genes, such as PRKCG and WNT1, exhibited HR less than 1, indicating they may serve as protective factors against MM, suggesting their expression is associated with a lower risk of disease or better prognosis. Conversely, 9 genes, including RAC3 and VANGL1, had HR values greater than 1, categorizing them as risk factors for MM, where their expression could indicate a higher risk of disease or poorer prognosis (*p* < 0.05, HR ≠ 1, Figure 1A). Subsequently, we performed consistency clustering on the selected 19 genes. The CDF curve plateaued when k reached the optimal number of clusters, verified by the Delta area, resulting in k = 2 (Figure 1B). Thus, we categorized all samples into two subtypes: C1 and C2, which exhibited characteristics of “high cohesion, low coupling” in the consistency heatmap (Figures 1C, D). We then conducted survival analysis on subtypes C1 and C2. The OS prediction for C1 was significantly higher than that for C2 (*p* < 0.05, Figure 1E).

### 3.2 Model construction and risk assessment

**FIGURE 2:**
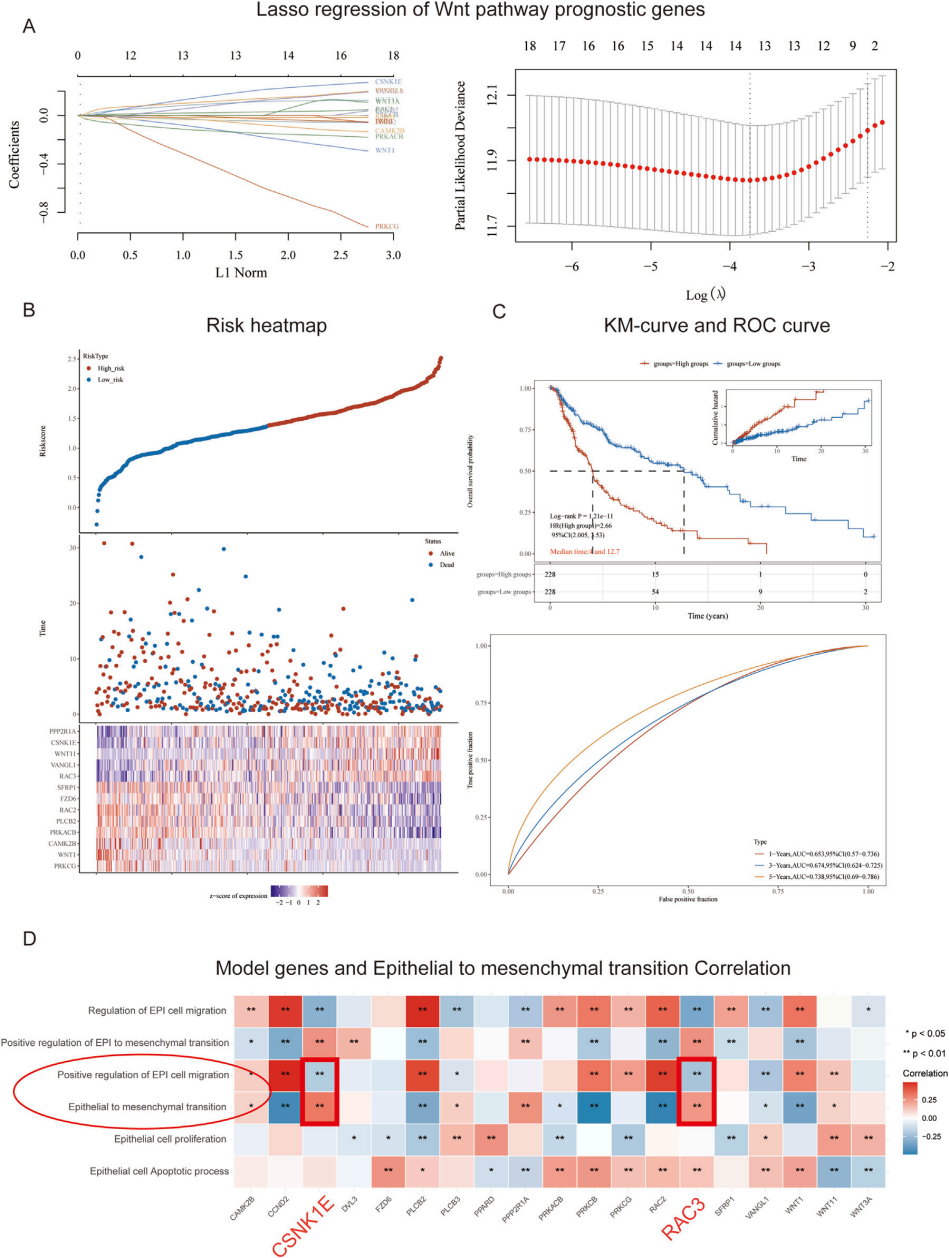
Model Construction and Risk Assessment. **(A)** Path plot of regression coefficients and cross-validation curve; **(B)** Triplet plot of risk scores for high and low-risk groups in the GSE91061 cohort; **(C)** Survival curves and ROC curves for high and low-risk groups in the GSE91061 cohort; **(D)** Heatmap showing the correlation between 13 genes and EMT.

We constructed a LASSO regression model using 19 selected genes. The cross-validation curve indicated that the optimal fitting effect was achieved when the variable corresponding to log (λ) was 13, as evidenced by the lowest point on the *y*-axis (Figure 2A). Basing on the median risk score, we stratified the GSE91061 dataset into high-risk and low-risk groups. Over time, both groups exhibited a significant decline in survival counts and an increase in mortality; however, the survival count in the high-risk group was markedly lower than that in the low-risk group. In the low-risk group, there’s an increase in the expression levels of SFRP1, FZD6, RAC2, PLCB2, PRKACB, CAMK2B, WNT1, and PRKCG, while in the high-risk group, PPP2RIA, CSNK1E, WNT11, VANGL1, and RAC3 showed higher expression levels (Figure 2B). Subsequently, we conducted survival analysis for both risk groups. As time progressed, the OS predictions for both groups declined, while cumulative risk increased. It is important to highlight that the overall survival predictions for the high-risk group were markedly lower than those for the low-risk group, which also showed a significantly lower cumulative risk. In order to evaluate the model’s predictive performance, we employed ROC curves, which revealed AUC values exceeding 0.6 for 1-year, 3-year, and 5-year predictions, indicating satisfactory predictive performance (Figure 2C). Furthermore, we analyzed the correlation between the model genes and physiological processes associated with EMT. Our findings demonstrated a negative correlation between the physiological process of positive regulation of epithelial cell migration and the genes CSNK1E and RAC3, while a positive correlation was observed between the epithelial-to-mesenchymal transition process and CSNK1E and RAC3 (*p* < 0.01, Figure 2D).

### 3.3 Survival analysis and immune correlation analysis

**FIGURE 3:**
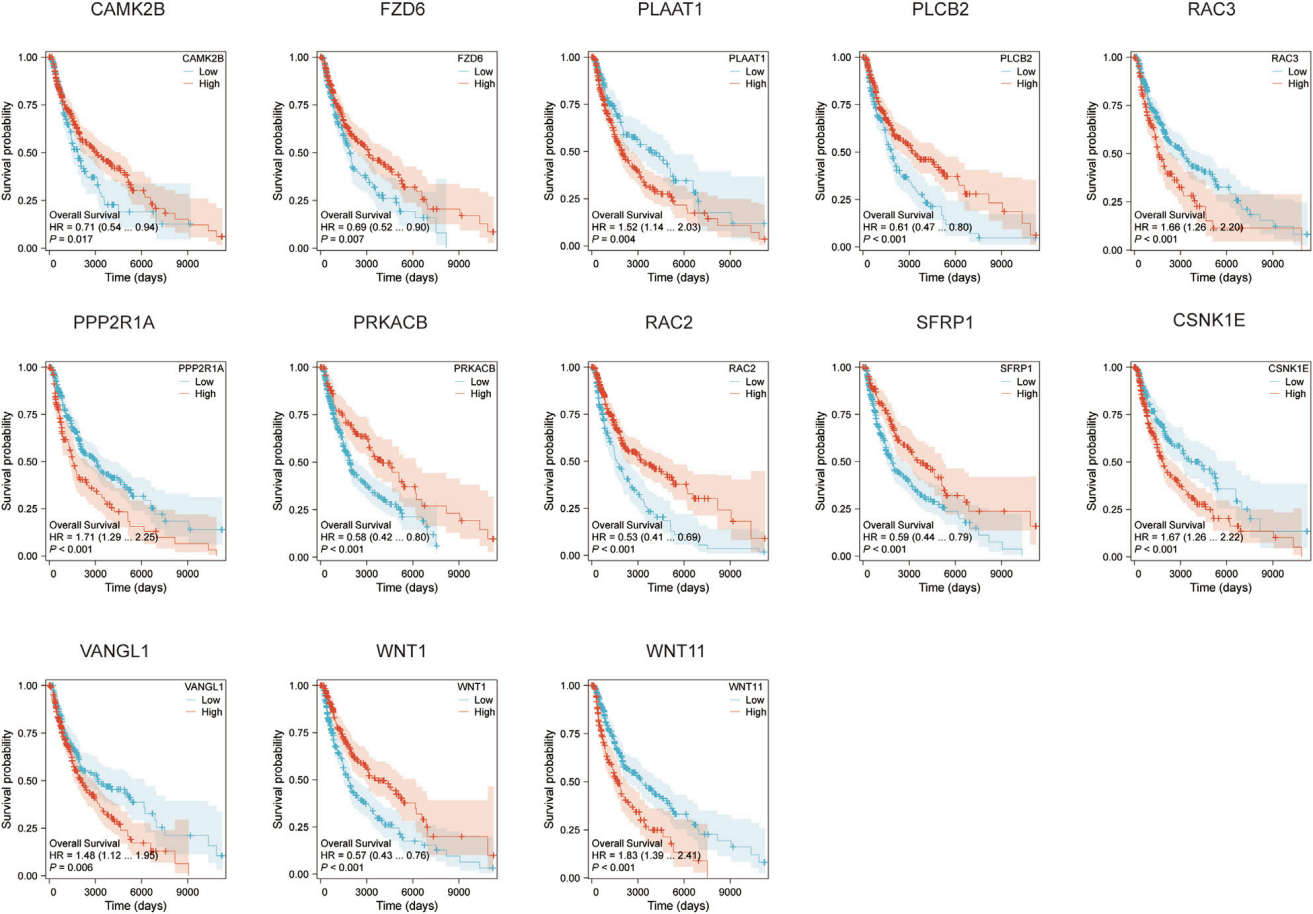
Survival curves for the 13 genes.

**FIGURE 4:**
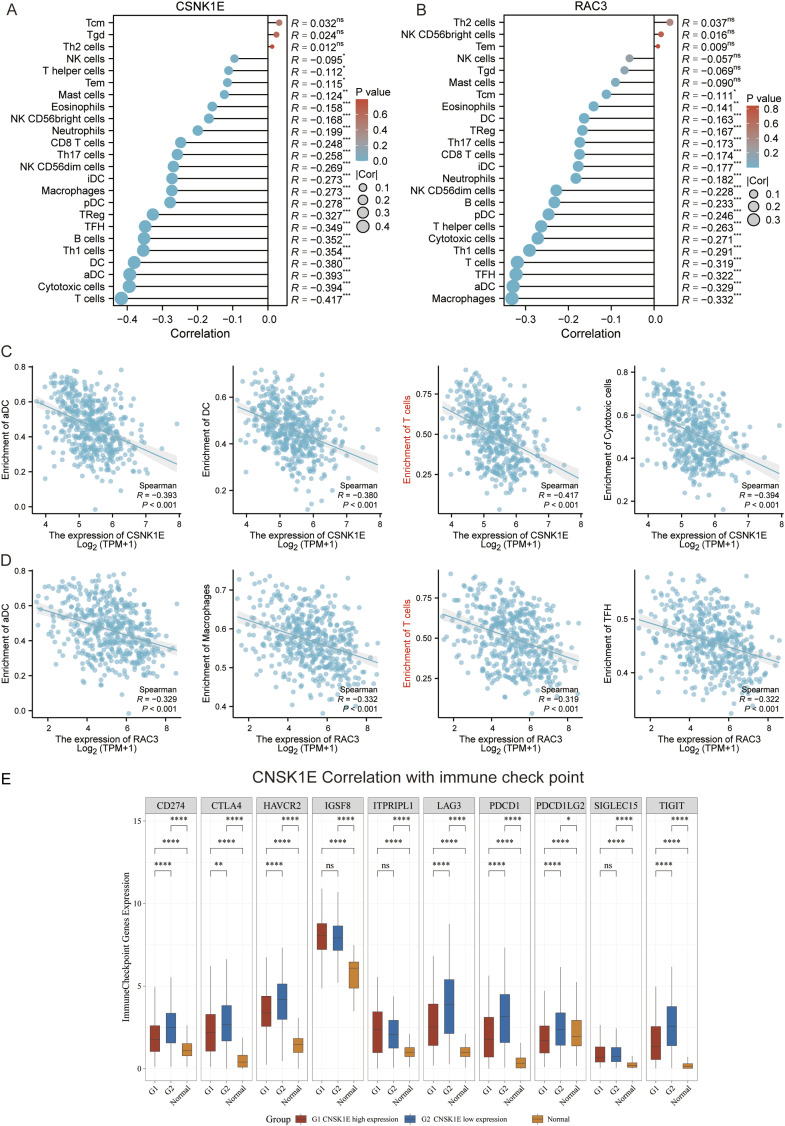
Immune-Related Analysis. **(A)** Lollipop plot depicting the correlation between CSNK1E and 24 immune cell types; **(B)** Lollipop plot depicting the correlation between RAC3 and 24 immune cell types; **(C)** Scatter plot illustrating the correlation between CSNK1E and four immune cell types; **(D)** Scatter plot illustrating the correlation between RAC3 and four immune cell types; **(E)** Boxplot of expression differences for 10 immune checkpoint genes associated with CSNK1E across high, low, and normal expression groups.

We conducted survival analyses on the 13 genes identified in our model, assessing the variations in survival rates associated with high versus low expression levels. Among these, the high expression group of CAMK2B, FZD6, PLCB2, PRKACB, RAC2, SFRP1, and WNT1 exhibited significantly better survival rates than the low expression group. In contrast, the low expression group of PLAAT1, RAC3, PPP2R1A, CSNK1E, VANGL1, and WNT11 was associated with significantly better survival rates (*p* < 0.05, Figure 3). Additionally, we conducted an analysis of the correlation between CSNK1E and RAC3 across 24 different immune cell types. CSNK1E demonstrated negative regulatory relationships with 21 immune cells, notably the strongest with T cells, while RAC3 showed similar negative correlations with 18 immune cells, particularly with Macrophages and activated dendritic cells (*p* < 0.05, R < 0, Figures 4A, B). We chose the four immune cell types that exhibited the strongest correlations with these genes for scatter plot analysis. This analysis revealed a negative correlation between CSNK1E expression and T cells, cytotoxic cells, activated dendritic cells, and dendritic cells (*p* < 0.001, R < −0.3, Figure 4C), as well as a similar relationship for RAC3 with Macrophages, activated dendritic cells, TFH, and T cells (*p* < 0.001, R < −0.3, Figure 4D). Notably, both genes exhibit a significant negative correlation with T cells. Subsequently, the relationship between CSNK1E expression and ten immune checkpoint genes was analyzed. Fluctuations in CSNK1E expression correlated with elevated levels of six immune checkpoints: CD274, CTLA4, HAVCR2, LAG3, PDCD1, and TIGIT, particularly pronounced in CNSK1E low expression group. In contrast, IGSF8, ITPRIPL1, and SIGLEC15 showed no significant differences between the high and low expression groups of CSNK1E; however, the expression levels in both groups were higher than those in the normal group. Elevated immune checkpoint expression suggests a stronger suppression of immune function, potentially linked to poorer prognoses in MM (*p* < 0.05, Figure 4E).

### 3.4 Pan-cancer analysis

**FIGURE 5:**
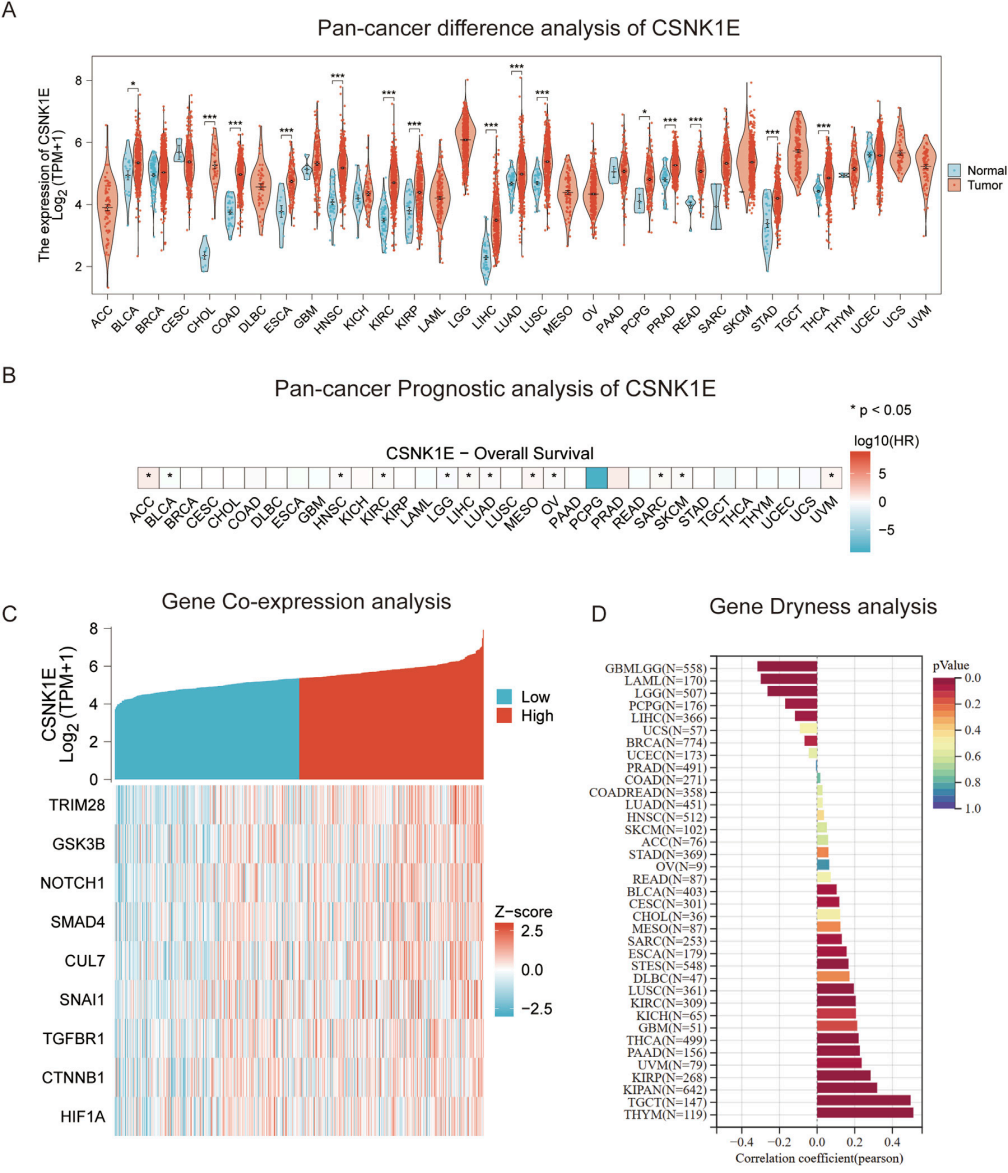
Pan-Cancer Analysis. **(A)** Violin plot of differential expression of CSNK1E across 33 cancer types; **(B)** Heatmap of survival analysis for CSNK1E in 33 cancer types; **(C)** Co-expression heatmap of CSNK1E with EMT-related genes across high and low expression groups; **(D)** Graphical representation of stemness analysis across 37 cancer types.

We conducted a pan-cancer analysis of CSNK1E across 33 different cancer types, comparing its expression levels in tumor versus normal groups. Notably, in 15 cancers, including bladder cancer (BLCA), cholangiocarcinoma (CHOL), colorectal cancer (COAD), esophageal cancer (ESCA), head and neck squamous cell carcinoma (HNSC), kidney renal clear cell carcinoma (KIRC), kidney renal papillary cell carcinoma (KIRP), liver hepatocellular carcinoma (LIHC), lung adenocarcinoma (LUAD), lung squamous cell carcinoma (LUSC), pan-cancer (PCPG), prostate cancer (PRAD), rectum adenocarcinoma (READ), stomach adenocarcinoma (STAD), and thyroid carcinoma (THCA), the tumor group exhibited significantly higher expression levels compared to the normal group (*p* < 0.05, Figure 5A). Subsequently, we performed a prognostic analysis across these 33 cancers, identifying CSNK1E as a risk factor in 12 cancer types, including adrenocortical carcinoma (ACC), BLCA, HNSC, KIRC, lower grade glioma (LGG), LIHC, LUAD, mesothelioma (MESO), ovarian cancer (OV), sarcoma (SARC), skin cutaneous melanoma (SKCM), and uveal melanoma (UVM), with a correlation to poorer prognosis (*p* < 0.05, log10(HR) > 0, Figure 5B). We further analyzed the co-expression of CSNK1E with genes related to EMT. In the low expression group, the levels of nine EMT-regulating genes—TRIM28, GSK3B, NOTCH1, SMAD4, CUL7, SNAI1, TGFBR1, CTNNB1, and HIF1A—were significantly diminished compared to those in the high expression group. This finding suggests a potential association between increased CSNK1E expression and enhanced EMT activity (Figure 5C). Finally, we assessed gene stemness across 37 cancers, observing a significant negative correlation in brain cancer (GBMLGG), acute myeloid leukemia (AML), and LGG, while a noteworthy positive correlation was found in both testicular cancer (TGCT) and thymoma (*p* < 0.05, Figure 5D).

### 3.5 CSNK1E plays a pro-cancer role in melanoma

**FIGURE 6:**
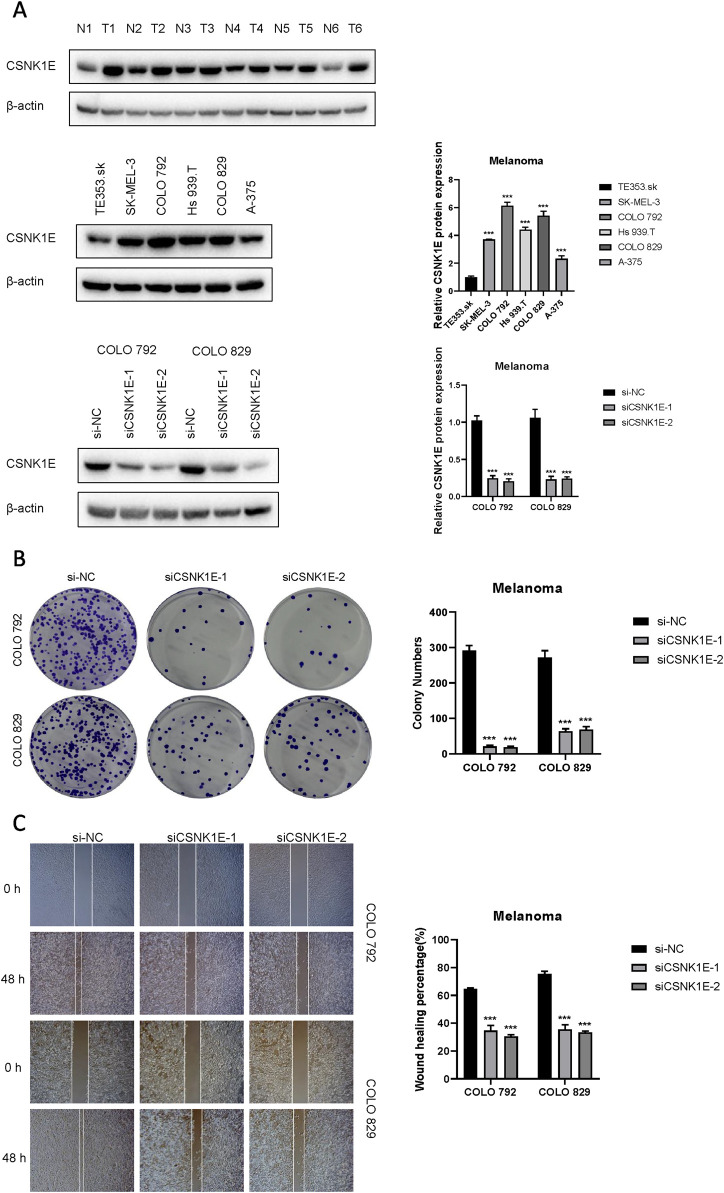
Expression of CSNK1E in various tissues and cell lines, and its impact on proliferation and migration. **(A)** Western blot images and corresponding bar graphs of relative protein expression levels of CSNK1E in different tissues and cell lines, as well as following knockdown; **(B)** Images from the colony formation assay along with the corresponding bar graph of colony numbers; **(C)** Images from the wound healing assay and bar graph showing the percentage of wound healing.

**FIGURE 7:**
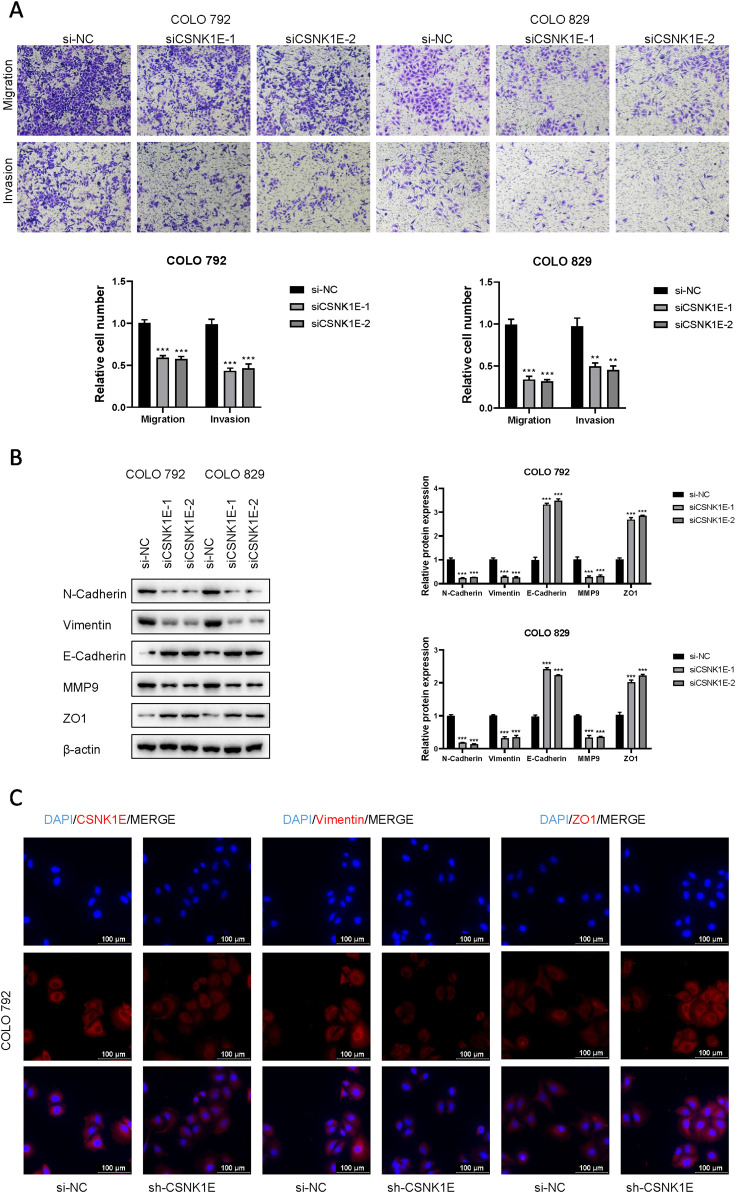
Effects of CSNK1E on invasion and migration, along with its influence on related protein expression. **(A)** Images from the transwell assay stained with crystal violet and bar graph of relative cell numbers; **(B)** Western blot images and corresponding bar graph of relative expression levels; **(C)** Immunofluorescence images.

**FIGURE 8:**
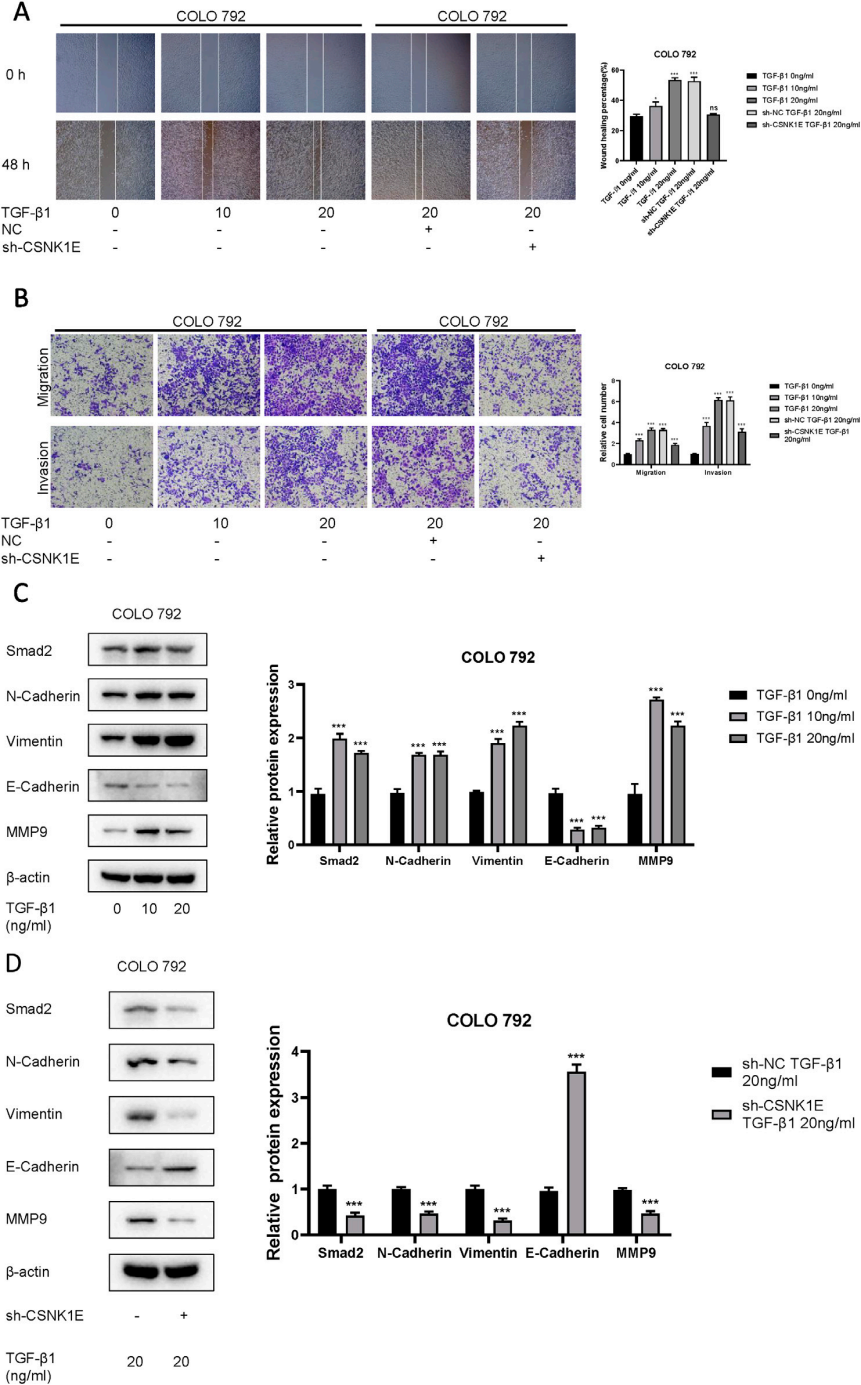
Investigation of the interaction between CSNK1E and TGF-β1. **(A)** Images from the wound healing assay and bar graph of wound healing percentages after CSNK1E knockdown at different concentrations of TGF-β1; **(B)** Images from the transwell assay stained with crystal violet and bar graph of relative cell numbers at varying concentrations of TGF-β1; **(C)** Western blot images and bar graph of relative expression levels of proteins in the COLO 792 cell line under different doses of TGF-β1; **(D)** Western blot images and bar graph of relative expression levels after CSNK1E knockdown at a fixed concentration of TGF-β1 in the COLO 792 cell line.

According to the results from Western blot assays, CSNK1E is expressed in both normal and tumor tissues, with significantly higher levels in tumor samples. In six cell lines—COLO 792, COLO 829, TE353. sk, SK-MEL-3, Hs 939. T, and A-375—CSNK1E expression was notably greater in cancer cell lines in comparison to normal skin cells. The bar graph depicting the relative expression levels of proteins indicates that the silencing of CSNK1E resulted in a marked decrease in protein levels, dropping below 50% of the NC group, indicating effective transfection (*p* < 0.001, Figure 6A). Images and corresponding bar graphs from the colony formation assays for COLO 792 and COLO 829 reveal that silencing CSNK1E significantly decreased the number of colonies formed, indicating reduced cell proliferation (*p* < 0.001, Figure 6B). Results from wound healing assays demonstrated a significant decrease in wound healing percentage after CSNK1E silencing, reflecting diminished cell migration capabilities (*p* < 0.001, Figure 6C). Transwell assays demonstrated that the number of invasive and migratory cells in the CSNK1E knockdown groups was significantly reduced compared to the NC group. Further indicating reduced invasive and migratory abilities (*p* < 0.01, Figure 7A). After silencing CSNK1E, there was a notable rise in the protein levels of E-Cadherin and ZO1, while levels of N-Cadherin, Vimentin, and MMP9 significantly decreased (*p* < 0.001, Figure 7B). Immunofluorescence results indicated that following shRNA-mediated CSNK1E knockdown, intracellular levels of CSNK1E and Vimentin decreased, while ZO1 levels increased (Figure 7C). Additionally, TGF-β1 was found to enhance cell migration, with varying effects at different doses. Specifically, as TGF-β1 concentration increased from 0 ng/mL to 10 ng/mL and 20 ng/mL, wound healing percentage significantly improved (*p* < 0.05). However, following CSNK1E knockdown, the migratory enhancement effect of TGF-β1 was diminished, showing no statistical difference in wound healing percentage at 20 ng/mL TGF-β1 compared to controls. This suggests that silencing CSNK1E can reverse the TGF-β1-induced migration in cancer cells, implying a potential synergistic role of CSNK1E in TGF-β1-related pathways (Figure 8A). Transwell experiments yielded similar results: increased TGF-β1 concentrations significantly enhanced cell invasion and migration, but the effects were attenuated following CSNK1E silencing, although the number of invasive and migratory cells at 20 ng/mL TGF-β1 remained higher than controls, indicating partial reversal of TGF-β1’s effects by CSNK1E knockdown (*p* < 0.001, Figure 8B). Finally, protein band and expression level bar graphs indicated that following TGF-β1 addition, E-Cadherin levels significantly decreased, while Smad2, N-Cadherin, Vimentin, and MMP9 levels increased. Notably, when TGF-β1 concentration increased from 10 ng/mL to 20 ng/mL, the levels of Smad2 and MMP9 decreased. Post-CSNK1E knockdown, only E-Cadherin levels significantly increased compared to controls, while the other four proteins showed significant reductions, further supporting the notion of CSNK1E’s synergistic role in TGF-β1-related pathways at the molecular level (*p* < 0.001, Figure 8C).

## 4 Discussion

Melanoma (MM) is the most lethal type of skin cancer and presents significant treatment challenges among solid tumors (Wolchok and Saenger, 2007). Its onset is linked to the malignant transformation of melanocytes (Lo and Fisher, 2014), and it exhibits a high level of immunogenicity, making immunotherapy a significant treatment modality. However, early immunotherapy approaches have shown substantial cytotoxicity (Ozbay Kurt et al., 2023). Data indicate that 40%–80% of patients may possess innate resistance to immune checkpoint inhibitors (ICIs), and the therapy combining CTLA-4 and PD-1 has been associated with severe adverse effects (Ballotti et al., 2020). Consequently, investigating the mechanisms underlying resistance in MM, identifying key biomarkers and exploring pivotal target genes are of great importance, as these factors are essential for diagnosis, treatment, and prognosis. There’s a close association between the WNT signaling pathway’s abnormal activation and the development and progression of several cancers (Zhang et al., 2018), including its role in promoting tumor dissemination and the development of resistance. Several proteins within the WNT pathway have been identified as potential therapeutic targets and biomarkers. However, research on the WNT pathway in MM remains limited. This study investigates the potential roles of WNT-related genes in MM and develops a prognostic model, thereby offering constructive insights and directions for discovering new therapeutic targets and enhancing prognosis in MM.

We conducted a Cox regression analysis on the gene set associated with the WNT pathway, identifying 19 genes linked to the occurrence and prognosis of malignant melanoma. Subsequently, these 19 genes underwent consistent clustering, resulting in two distinct subtypes. The consistency heatmap demonstrated characteristics of “high cohesion and low coupling”. The survival differences between clusters C1 and C2 were statistically significant, indicating the reliability of the classification and establishing that k = 2 is the optimal number of clusters. Next, we developed a LASSO regression model incorporating 13 of the identified genes, including PPR2R1A, CSNK1E, and WNT11. Using the risk scores generated by this model, we classified the samples into high-risk and low-risk groups. The high-risk group exhibited poorer survival outcomes, characterized by elevated expression of five genes, including CSNK1E and RAC3, suggesting their potential influence on prognosis. There was a statistically significant difference in survival between the high- and low-risk groups, with the low-risk group exhibiting a substantially higher survival rate. The ROC curve analysis revealed that the model demonstrated strong predictive performance, indicating that the results might be widely applicable. Additionally, we examined the relationship between the 13 genes and the EMT process. Notably, CSNK1E and RAC3 demonstrated a significant correlation with EMT, indicating their potential involvement in mediating the metastatic process of MM cells, as EMT facilitates tumor cell invasion through the basement membrane into the bloodstream. Survival analysis of the 13 genes revealed that high expression levels of CAMK2B, FZD6, PLCB2, PRKACB, RAC2, SFRP1, and WNT1 were associated with improved survival rates, suggesting that the activation of these genes may positively influence MM prognosis. Conversely, the activation of PLAAT1, RAC3, PPP2R1A, CSNK1E, VANGL1, and WNT11 correlated with poorer prognostic outcomes.

We assessed the relationship between CSNK1E and RAC3 across 24 immune cell types, discovering that CSNK1E had the strongest correlation with T cells, whereas RAC3 was most closely linked to macrophages and also showed a notable association with T cells. Considering the crucial impact of immune checkpoint expression on T cell functionality, we further explored the connection between CSNK1E and ten immune checkpoints.

We then performed a pan-cancer analysis of CSNK1E across 33 different cancer types. Remarkably, in 15 of these cancers, including BLCA, CSNK1E expression levels in tumor samples were significantly elevated compared to normal tissues. This finding implies that CSNK1E might have a comparable role in various cancers, leading us to propose that it could act as a potential biomarker for early diagnosis and treatment across multiple cancer types. Additionally, we investigated the relationship between CSNK1E expression and prognosis across the 33 cancer types, identifying it as a risk factor in 12 of them, including ACC and BLCA. Following this, we analyzed the co-expression patterns of CSNK1E with genes linked to EMT. The high-expression group of CSNK1E exhibited more active expression of EMT-related genes, leading us to speculate that CSNK1E may influence the invasiveness and metastatic capabilities of MM cells through its regulatory role in EMT. This could potentially provide new therapeutic targets for MM treatment and prognosis assessment. Finally, we explored the relationship between stem cells and 37 cancer types. Our findings may offer a novel target for MM therapy and provide theoretical support for advancements in biotechnology.

Using the LASSO machine learning algorithm, we identified 19 genes associated with the WNT signaling pathway and constructed a regression model comprising 13 genes, including PRR2R1A, CSNK1E, WNT11, VANGL1, and RAC3. The CSNK1E gene encodes casein kinase 1 epsilon (CK1ε), which primarily regulates circadian rhythms by phosphorylating clock gene products (Knippschild et al., 2005). Additionally, CK1ε influences cell differentiation and proliferation through protein phosphorylation (Meng et al., 2010). Moreover, CK1ε is capable of phosphorylating other critical proteins within the WNT signaling pathway, so that it can regulate cell division and tumor growth in pancreatic cancer, salivary gland cancer, and colorectal adenocarcinoma (Brockschmidt et al., 2008; Frierson et al., 2002). For example, CK1ε is involved in phosphorylating low-density lipoprotein receptor-related proteins 5 and 6 (LRP5/6) as well as Dvl (Price, 2006), which subsequently promotes the recognition of the Axin and glycogen synthase kinase 3 beta (GSK-3β) complex (Mao et al., 2001). The phosphorylation of β-catenin by GSK-3β is inhibited by the LRP5/6 complex subsequently (Piao et al., 2008), thereby extending the half-life of β-catenin (Del Valle-Pérez et al., 2011). Additionally, CK1ε collaborates with GSK3β to phosphorylate adenomatous polyposis coli (APC), thereby facilitating the binding of β-catenin to APC (Rubinfeld et al., 2001). In the p53 signaling pathway, DNA damage facilitates the interaction between CK1ε and its binding partner, MDM2, resulting in multivalent phosphorylation of MDM2 and enhancing p53 activity (Schittek and Sinnberg, 2014). Nonetheless, there is a notable absence of literature on the role of CSNK1E in MM at present, highlighting the need for further research into its functions in tumor biology. RAC, belonging to the Rho GTPase subfamily (Hodge and Ridley, 2016), encompasses three isoforms: RAC1, RAC2, and RAC3 (Haataja et al., 1997). These proteins, along with their closely related homolog Cdc42, play multifaceted roles in cellular processes such as cytoskeletal regulation, EMT, transcription, proliferation, cell polarity, apoptosis, phagocytosis, and vesicular transport. They serve as central regulatory factors in the metastasis and invasion of cancer cells (Maldonado et al., 2020). Notably, overexpression of RAC3 has been implicated in the development of various cancers. In typical circumstances, RAC3 is mainly found in brain tissue and neuronal cells (Corbetta et al., 2009), yet its expression is upregulated in breast cancer, prostate cancer, and brain tumors. In aggressive breast cancer, RAC3’s specific binding partner CIB1 facilitates the recruitment of RAC3, promoting integrin activation at invasive pseudopodia, thereby regulating adhesion and degradation of the extracellular matrix (ECM) (Wang et al., 2022). With its ectopic expression allowing cells to avoid excessive autophagy and cell death caused by the inhibition of isoprenylcysteine carboxyl methyltransferase (Icmt) (Zhu et al., 2011). Nevertheless, the precise function of RAC3 in MM is still not well defined, highlighting the necessity for further research into its roles.

## 5 Conclusion

In this study, we first conducted Cox regression analysis on a gene set associated with the WNT signaling pathway, followed by consistent clustering. We then employed the LASSO algorithm to construct a model and assessed risk within the GSE91061 cohort. Additionally, we examined the relationship between 13 genes and EMT, conducting immune analysis on the two genes that showed the strongest correlations. Finally, a pan-cancer analysis of CSNK1E was conducted, and we explored the co-expression of EMT-related genes. Our findings offer new targets for MM research, providing theoretical support for both scientific inquiry and clinical investigation.

## Data Availability

Publicly available datasets were analyzed in this study. This data can be found here: Raw data and WB original images: https://www.jianguoyun.com/p/DSolhzMQ9rv3DBiSieQFIAA.
